# Morphological and life-history responses of anurans to predation by an invasive crayfish: an integrative approach

**DOI:** 10.1002/ece3.979

**Published:** 2014-03-25

**Authors:** Ana L Nunes, Germán Orizaola, Anssi Laurila, Rui Rebelo

**Affiliations:** 1 Centro de Biologia Ambiental, Departamento de Biologia Ambiental, Faculdade de Ciências da Universidade de LisboaLisbon, Portugal; 2 Animal Ecology/Department of Ecology and Genetics, Evolutionary Biology Centre, Uppsala UniversityUppsala, Sweden

**Keywords:** Crayfish, integration of responses, invasive species, phenotypic plasticity, tadpoles

## Abstract

Predator-induced phenotypic plasticity has been widely documented in response to native predators, but studies examining the extent to which prey can respond to exotic invasive predators are scarce. As native prey often do not share a long evolutionary history with invasive predators, they may lack defenses against them. This can lead to population declines and even extinctions, making exotic predators a serious threat to biodiversity. Here, in a community-wide study, we examined the morphological and life-history responses of anuran larvae reared with the invasive red swamp crayfish, *Procambarus clarkii*, feeding on conspecific tadpoles. We reared tadpoles of nine species until metamorphosis and examined responses in terms of larval morphology, growth, and development, as well as their degree of phenotypic integration. These responses were compared with the ones developed in the presence of a native predator, the larval dragonfly *Aeshna* sp., also feeding on tadpoles. Eight of the nine species altered their morphology or life history when reared with the fed dragonfly, but only four when reared with the fed crayfish, suggesting among-species variation in the ability to respond to a novel predator. While morphological defenses were generally similar across species (deeper tails) and almost exclusively elicited in the presence of the fed dragonfly, life-history responses were very variable and commonly elicited in the presence of the invasive crayfish. Phenotypes induced in the presence of dragonfly were more integrated than in crayfish presence. The lack of response to the presence of the fed crayfish in five of the study species suggests higher risk of local extinction and ultimately reduced diversity of the invaded amphibian communities. Understanding how native prey species vary in their responses to invasive predators is important in predicting the impacts caused by newly established predator–prey interactions following biological invasions.

## Introduction

Invasive species can modify ecosystems through habitat alteration, introduction of new diseases, genetic introgression, competition, and predation, and are considered one of the most serious ecological threats to biodiversity (Lockwood et al. 2007). By creating new interactions with native species, invasive species may profoundly alter ecosystems. Invasive predators have drastically reduced or extirpated populations of native species, largely due to a lack of coevolutionary history between predators and prey (reviewed in Kats and Ferrer 2003; Cox and Lima 2006). When a predator invades a new area, native prey may not be able to detect or identify the novel predator as a dangerous threat, resulting in lack of or ineffective antipredator responses (Cox and Lima 2006; Sih et al. 2010). This lack of native prey responses to invasive predators is likely to increase prey mortality and severely impact invaded populations and communities (e.g., Rodda et al. 1997). On the other hand, prey that have co-occurred with a predator in nature and share a common evolutionary history are usually able to detect and evaluate predation risk and use adequate defensive mechanisms that enhance their survival (Lima and Dill 1990; Sih et al. 2010).

Predator-induced defenses are a widespread form of phenotypic plasticity and involve alterations in behavior, morphology, physiology, and life history (e.g., Lima and Dill 1990; Benard 2004). Morphological defenses typically consist of developing structures, such as spines and helmets in crustaceans, alterations of body shape in fish and amphibian larvae, and thicker shells in mollusks, all of which improve survival under predation risk (e.g., Spitze 1992; Relyea 2001; Hoverman et al. 2005; Johansson and Andersson 2009). Predators can also induce alterations in the life history of their prey (Benard 2004; Beckerman et al. 2007; Relyea 2007). Predation risk can affect life-history decisions such as the time and the size at which key life-history switch points occur, which can strongly influence individual fitness (Benard 2004; Relyea 2007). For instance, reaching a life-history switch point faster can be a direct response to predation risk that allows prey to leave the risky environment earlier, thereby reducing mortality risk (Benard 2004; Higginson and Ruxton 2009). As large size may provide a refuge from predation, increasing growth rate can also be a direct and adaptive response to predation by gape or size-limited predators (Urban 2007b). Importantly, however, the growth/predation risk trade-off is a common constraint documented for many organisms, with higher growth rates coming at the expense of increased vulnerability to predators (e.g., Lima and Dill 1990; McPeek 2004).

An integrated view of inducible defenses states that selection acts in favor of expressing simultaneously multiple defensive traits (DeWitt and Langerhans 2003; Steiner and Pfeiffer 2007). Phenotypic integration is reflected in correlations between traits at genetic, developmental, and functional levels (Cheverud 1982), and the degree of their integration depends on the magnitude of the functional relationships among them (Relyea 2001; Hoverman et al. 2005). Behavioral and morphological defenses, as well as life-history trait alterations, are not just direct and independent responses to predation, but may occur together and often depend on each other, promoting phenotypic integration (Steiner 2007). Further, some of these relations may reflect trade-offs among traits, indicating fitness costs or benefits associated with the development of inducible defenses (e.g., Van Buskirk 2000; Relyea 2001; Higginson and Ruxton 2009). For example, behaviorally and morphologically defended prey may allocate fewer resources into growth and development, often resulting in reduced body size or delayed time to a life-history switch point (Spitze 1992; Van Buskirk 2000; Higginson and Ruxton 2010; but see Steiner 2007). However, behavioral and morphological defenses may also be linked with increased growth rates, indicating that the link between these defenses and growth rate is not necessarily straightforward (Spitze 1992; Peacor 2002; McPeek 2004; Johansson and Andersson 2009). The type and magnitude of relationships between different defensive traits vary across species and populations and can depend on the ecological context (Relyea 2001; DeWitt and Langerhans 2003; Higginson and Ruxton 2009). For instance, if exposed to a new predator, prey may show more weakly integrated defensive phenotypes than in the presence of a known predator, due to the lack of or shorter coevolutionary history selecting for a more integrated antipredator response.

Amphibian larvae provide an excellent system for examining antipredator responses to predators. In amphibians, larval predation is one of the major sources of mortality (Werner 1991), and many amphibians have been negatively impacted by invasive predators (Kats and Ferrer 2003). Lacking appropriate defenses towards a newly arrived predator may render native prey populations at serious risk; however, species within a community may greatly vary in their ability to detect and respond to novel predators. For instance, species that use more general – as opposed to predator-specific – chemical signals to assess predation risk are more likely to respond to the activity of novel predators (Sih et al. 2010; Nunes et al. 2013). In amphibians, examples of these signals are chemical alarm cues released by injured or consumed conspecifics (Sih et al. 2010). To date, several studies have examined behavioral responses to invasive predators in amphibians (e.g., Pearl et al. 2003; Polo-Cavia et al. 2010). However, only few have investigated morphological and life-history responses (e.g., Moore et al. 2004; Gómez-Mestre and Díaz-Paniagua 2011), and none of these have used a community-wide approach.

The red swamp crayfish *Procambarus clarkii*, endemic to northeastern Mexico and southcentral USA, is a successful invader worldwide (Holdich et al. 2009). It was introduced in Spain in 1973 and has now populations established all over the Iberian Peninsula (Holdich et al. 2009). Several studies have documented its strong negative impacts on European amphibians (Cruz et al. 2006; Ficetola et al. 2011). A recent community-wide study in southwestern Portugal showed that the tadpoles of five of nine anuran species elicited behavioral defenses when exposed to *P. clarkii*, while eight of the nine species responded to a common native predator (*Aeshna* sp. dragonfly larva; Nunes et al. 2013). The present work represents a second step of that study by investigating if tadpoles in this community show predator-induced plasticity in morphology and life-history traits when exposed to the crayfish actively preying on conspecific tadpoles. We also examined these responses when larvae were exposed to a native dragonfly larva also feeding on conspecific tadpoles. Further, we explored whether patterns of integration in responses differed in the presence of the native and newly arrived predators.

The aim of this study was not to directly compare responses between a native dragonfly and an invasive crayfish as the two predators differ in many aspects. Instead, the dragonfly is used as a positive control to reveal the tadpole responses to an actively feeding common predator. Both crayfish and dragonfly larvae consume tadpoles by piercing and chewing them, likely creating similar alarm cues. Consequently, tadpoles responding to the dragonfly but not to the crayfish are likely mainly responding to cues associated with the predator itself, either kairomones or cues generated during the digestion, and to a smaller extent to the cues from the consumed tadpoles. Responses to the crayfish could be associated either to general alarm cues, to specific cues produced by the predator, or to both. Importantly, this result would indicate that tadpoles can respond to a relatively new invasive predator that is feeding upon conspecific tadpoles.

We make the following predictions: (1) Most of the studied species will alter their morphology and life history when reared in the presence of the fed dragonfly. This is because many previous studies have reported that amphibian larvae show plastic morphological and/or life-history antipredator responses when exposed to chemical stimuli of active larval dragonflies (Van Buskirk 2002; Nicieza et al. 2006; Richter-Boix et al. 2007; Gómez-Mestre and Díaz-Paniagua 2011); (2) fewer species will show responses to the crayfish due to the shorter coevolutionary history with this predator; (3) species known to use general alarm cues to respond to predators (*Alytes cisternasii*,*Bufo bufo,* and *Discoglossus galganoi*; Nunes et al. 2013) will be more likely to show morphological or life-history alterations when reared in the presence of the newly arrived crayfish; (4) the species responding morphologically to the crayfish may develop deeper tails when reared with fed crayfish, because Gómez-Mestre and Díaz-Paniagua (2011) showed that this morphological alteration (induced by dragonflies) is effective in decreasing mortality of *Pelophylax perezi* exposed to *P. clarkii*; and (5) the patterns of phenotypic integration will vary across species, stronger patterns of integration likely appearing in tadpoles reared with dragonflies than with the invasive crayfish.

## Materials and Methods

### Study species and experimental procedure

The study area was located in the Sado River basin (southwest Portugal), characterized by a variety of freshwater habitats, ranging from streams to rice fields (Cruz et al. 2006). This area, which naturally lacks native freshwater crayfishes, was invaded by *P. clarkii* ca. 25 years ago (R. Rebelo, pers. obs.). The only native crayfish in Portugal, *Austropotamobius pallipes*, is currently extinct across the Portuguese territory, and its original geographical range was in central and northern Portugal, several hundred kilometers away from our study area (Almaça 1991). Consequently, none of the amphibian populations used in this study has been in contact with a native crayfish predator.

Nine anuran species occur in this area: Iberian midwife toad (*Alytes cisternasii*), Iberian painted frog (*Discoglossus galganoi*), western spadefoot toad (*Pelobates cultripes*), Iberian parsley frog (*Pelodytes ibericus*), common toad (*Bufo bufo*), natterjack toad (*Bufo calamita*), European tree frog (*Hyla arborea*), Mediterranean tree frog (*Hyla meridionalis*) and Iberian water frog (*Pelophylax perezi*). Three of these species are Iberian endemics (*A. cisternasii*,*D. galganoi,* and *P. ibericus*) and three others (*P. cultripes*,*H. meridionalis,* and *P. perezi*) have a restricted distribution outside Iberia (Loureiro 2008). *P. clarkii* is an efficient predator of larvae of all these species and is now abundant throughout southwestern Portugal (Cruz and Rebelo 2005). The native predator used in this study, late-instar dragonfly larvae (*Aeshna* sp.), is widespread in the study area. Both these predators co-occur with all the anuran species used in this study, imposing a constant high predation pressure on tadpoles. Other potential predators of tadpoles present in the study area are dytiscid and anisopteran larvae, heteropterans, as well as some vertebrates (mainly birds and snakes).

Because of differences in breeding phenology among the species, experiments were conducted in different times throughout the year. From November 2007 to June 2008, according to species phenology, several clutches of eight of the nine anuran species were collected in streams or ponds in the study area (Table 1). For *A. cisternasii*, a species in which males carry the eggs until hatching, larvae were collected very shortly after being released in the water. A reproducing *P. clarkii* population was present in all the water bodies where egg masses were collected. The egg clutches were transported to the field station of the Centre for Environmental Biology in Grândola (38º06′N, 8º34′W), where the experiment took place. Embryos and larvae were kept in several species-specific 5L containers filled with spring water that was changed every 3 days and were fed commercial fish food and boiled lettuce ad libitum. After reaching Gosner stage 25 (operculum closure over gills; Gosner 1960), they were randomly assigned to different treatments, and the experiment was initiated. Adult crayfish and late-instar larval dragonflies were caught in ponds close to the field station (<30 km). While not in the experiment, crayfishes were kept in several 40-L plastic tanks and fed commercial fish food and small invertebrates, whereas dragonfly larvae were kept individually in 1.2-L plastic boxes and fed *Ephemeroptera* larvae. Prior to entering the experiment, predators were starved for 48–72 h.

**Table 1:** Information on collection and experimental procedures for the nine studied species.

Species	Nr. clutches collected	Date of collection	Start of experiment	Morphology registered	Gosner stage	End of experiment
*Alytes cisternasii*	–	04 December	15 December	12 February	30	28 July
*Discoglossus galganoi*	9	08 January	24 January	26 February	35	13 May
*Pelobates cultripes*	8	28 November	15 December	16 April	30	05 September
*Pelodytes ibericus*	10	26 December	23 January	20 February	32	22 June
*Bufo bufo*	6	07 February	19 February	20 March	34	04 June
*Bufo calamita*	9	22 April	19 May	17 June	34	09 August
*Hyla arborea*	10	05 June	17 June	23 July	30	25 September
*Hyla meridionalis*	9	13 March	08 April	20 May	32	15 August
*Pelophylax perezi*	11	16 June	02 July	03 September	33	09 November

We performed a factorial experiment using nine anuran species and three predator treatments: dragonfly, crayfish, and control (i.e., no predators). At the beginning of the experiment, groups of ten tadpoles were allocated to plastic containers (39 × 28 × 28 cm) filled with 10 L of water. A cylindrical predator cage, containing either one crayfish (cage diameter 85 mm, length 150 mm), one dragonfly larva (cage diameter 62 mm, length 130 mm) or no predator (randomly one of the two sizes) was added to each container. Cages were opaque and covered with fine mesh netting on both sides, so that chemical cues could flow out, but visual or tactile cues were not available. This design was selected because chemical cues from predatory events are known to elicit a full suite of antipredator responses in aquatic organisms (Ferrari et al. 2010). Predators were fed three tadpoles conspecifics to the experimental individuals every second day. For each of the nine species, each of the three treatments was replicated five times, once in each of five randomized blocks, resulting in a total of 135 experimental units. Once tadpoles approached metamorphosis (Gosner stage 42: emergence of at least one forelimb), we checked the containers daily for metamorphosed individuals, which were then removed. Throughout the experiment, the tadpoles were fed fish flakes and boiled lettuce ad libitum three times per week and water was changed every 5 days. Water temperature was 18.0 ± 0.17°C (mean ± SE) and the photoperiod 12L:12D.

### Response variables

In order to examine tadpole morphology, five haphazardly selected tadpoles per container were photographed with a digital camera in side view against a grid background. Photographs were taken, depending on species, when tadpoles were between Gosner developmental stages 30 and 35 (see Table 1). Images were loaded into MakeFan7 (Sheets 2009) to create a standardized template for digitizing the different landmarks. The body shape of tadpoles was captured by digitizing 20 landmarks on each individual (Fig. S1) using tpsDig2 software (Rohlf 2008). Some of the landmarks were chosen according to specific and easily identifiable anatomical points in the tadpole body (e.g., the center of the eye, the tip of the tail muscle and fin), while others were defined using proportional distances within a structure (Fig. S1). We conducted landmark-based geometric morphometrics analyses on these digitized landmarks; this method is a powerful tool for analyzing and visualizing morphological variation between individuals (Rohlf and Marcus 1993; Zelditch et al. 2004). Digitized landmarks were imported into tpsRelw (Rohlf^2007^), where a generalized least-squares Procrustes analysis was performed in order to standardize the size, translate and rotate the configurations of landmark coordinates (Rohlf and Slice 1990). We used the same software to extract relative warp scores, which were then used as shape variables in the analysis. Relative warp scores were estimated using the consensus (i.e., average) morphology for each experimental container in order to avoid pseudoreplication. Consensus conformations were estimated using tpsRelw (Rohlf 2007).

At metamorphosis, tadpoles were blotted dry and weighed with a digital balance to the nearest 0.01 g (mass at metamorphosis). Time to metamorphosis was defined as the number of days elapsed between the beginning of the experiment (day of Gosner stage 25) and the onset of metamorphosis (day of Gosner stage 42). Growth rate was estimated as the quotient between mass at and time to metamorphosis.

### Statistical analyses

**Figure 1:**
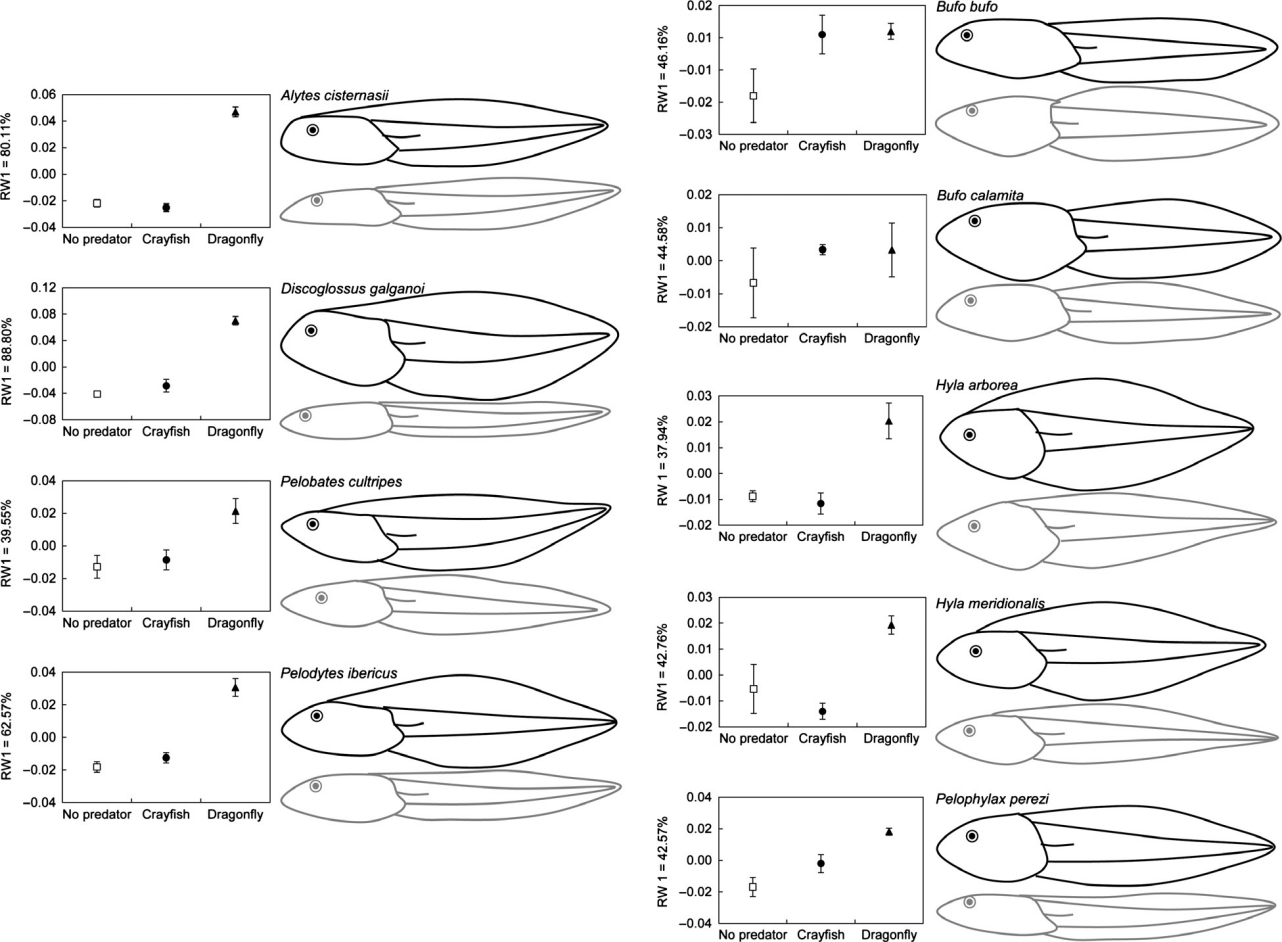
Mean ± SE values of relative warp 1 (RW1) representing tadpole shape of the nine anuran species in the presence of different predator treatments. Drawings placed on the right-hand side of each graph show the shape of larvae representing the extreme positive (black) and negative (gray) scores of RW1.

All morphological analyses were conducted on relative warp scores and independently for each of the nine studied species. The body shape of tadpoles was examined using the first relative warp yielded by the geometric morphometrics analyses (RW1) which accounted, on average, for over 50% of the total morphological variance (Fig. 1; see Orizaola et al. (2012) for a similar approach). We analyzed the effects of the different predator treatments on tadpole morphology using univariate analyses of variance (ANOVAs) with type III sum of squares, where RW1 scores were the dependent variable describing tadpole morphology. Post hoc analyses were performed using Tukey HSD tests.

To investigate the effects of predator treatments in larval life-history traits (time to metamorphosis, dry body mass at metamorphosis, and growth rate), we performed univariate ANOVAs with type III sum of squares for each species and trait. Tank means were always used as the unit of analysis, and values were log-transformed in order to fulfill the assumptions of analysis of variance. Block effects were not significant for any of the traits and were excluded from the analyses. Post hoc comparisons were performed using Tukey HSD tests. An analysis comparing all species was not performed, as species responses were expected to differ, and we were mostly interested in examining whether and how a specific species was responding to the crayfish.

To examine associations between different antipredator responses and their phenotypic integration, we started by determining whether predator-induced changes in morphology were associated with induced changes in life-history characters. We also used behavioral data from the same study published elsewhere (Nunes et al. 2013) to investigate relationships between alterations in behavior (the average proportion of active tadpoles across ontogeny for each species), morphology, and life-history traits. These associations were tested by performing Pearson correlations between different traits, on tank means, for each species separately and across predator environments. However, as we were interested in understanding whether the presence of crayfish induced associations and patterns of integration different than the ones induced by the native dragonfly, correlations were performed separately for each predator plus the control treatment (crayfish + control or dragonfly + control; *N* = 10 per species and trait pair). The degree of phenotypic integration expressed by each species in response to each predator environment was considered to be higher as the number and magnitude of significant correlations among traits increased (Relyea 2001; Hoverman et al. 2005). Across-species correlations were not performed because we were interested in looking at associations and integration of responses within each species.

## Results

### Morphological responses

Values of relative warp 1 (RW1) usually increased in the presence of fed predators, and high positive scores for RW1 generally describe bulgier tadpoles, having relatively deeper headbodies and tails and a more anterior insertion of the tail fin into the headbody (Fig. 1). All species except *B. calamita* modified their morphology when reared with the fed dragonfly larvae (Table 2; Fig. 1). Of these eight species, all except *B. bufo* developed deeper tail fins, and all but *B. bufo* and *P. perezi* had a more anterior insertion of the tail fin to the headbody (Fig. 1). Induced tadpoles of *A. cisternasii*,*D. galganoi*,*P. perezi,* and *P. cultripes* also had deeper tail muscles. The three former species together with *P. ibericus* and *H. meridionalis* also had deeper headbodies (Fig. 1). *B. bufo* altered its morphology by developing a longer headbody (Fig. 1). On the contrary, when reared with the fed crayfish, only *B. bufo* changed its morphology, the response being similar to that found in the presence of the dragonfly (Table 2; Fig. 1).

**Table 2:** Univariate ANOVAs on the morphology (first relative warp, RW1) of the nine anuran species in presence of different predator treatments (left-hand panel). *P*-values for the Tukey post hoc tests referring to comparisons between the control and each predator treatment (right-hand panel). *P*-values < 0.05 are marked in boldface.

	Morphology			Dragonfly	Crayfish
	df	*F*	*P*	*P*	*P*
*Alytes cisternasii*	2, 15	165.786	**<0.001**	**<0.001**	0.745
*Discoglossus galganoi*	2, 15	79.434	**<0.001**	**<0.001**	0.419
*Pelobates cultripes*	2, 15	7.207	**0.009**	**0.012**	0.907
*Pelodytes ibericus*	2, 15	42.452	**<0.001**	**<0.001**	0.610
*Bufo bufo*	2, 15	28.615	**<0.001**	**<0.001**	**0.005**
*Bufo calamita*	2, 15	0.121	0.887	–	–
*Hyla arborea*	2, 15	13.654	**0.001**	**0.003**	0.910
*Hyla meridionalis*	2, 15	8.047	**0.006**	**0.035**	0.589
*Pelophylax perezi*	2, 13	14.796	**0.001**	**0.001**	0.164

### Life-history responses

**Figure 2:**
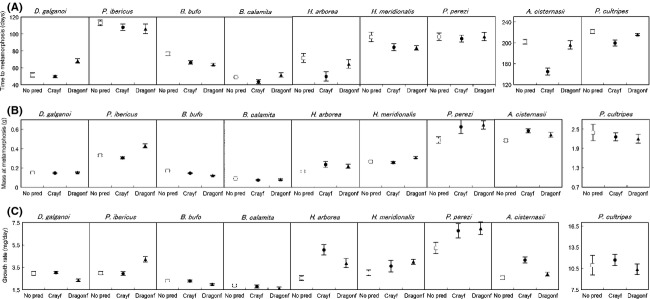
(A) Time to metamorphosis, (B) mass at metamorphosis and (C) growth rate (mean ± SE) of the nine anuran species under different predator treatments. The three treatments are control (No pred), crayfish (Crayf), and dragonfly (Dragonf). Note different scales in the graphs at the end of each row for species having very high values of a specific trait.

Five species altered their life-history traits when reared with dragonfly larvae: *B. bufo*,*D. galganoi*,*H. arborea*,*H. meridionalis,* and *P. ibericus* (Table 3). Induced tadpoles of *P. ibericus* increased growth rate and metamorphosed at larger mass (Table 3; Fig. 2). Tadpoles of *H. meridionalis* attained a larger mass at metamorphosis, while *H. arborea* tadpoles increased their growth rates. On the contrary, *B. bufo* tadpoles decreased both growth rate and time to metamorphosis, which resulted in a smaller mass at metamorphosis (Table 3; Fig. 2). Tadpoles of *D. galganoi* also reduced their growth rates, but they took longer to metamorphose; this was the only species to increase time to metamorphosis in response to fed predator presence (Table 3; Fig. 2).

**Table 3:** Analyses of life-history traits (time to metamorphosis, mass at metamorphosis, growth rate) of the nine anuran species. Results (*P*-values) of the univariate ANOVAs performed for each trait (df = 2, 15) are shown on the left-hand side of each column. Results (*P*-values) of the Tukey post hoc tests are shown on the right-hand side; values refer to comparisons between each predator and the control treatments. *P*-values < 0.05 are marked in boldface.

	Time to metamorphosis			Mass at metamorphosis			Growth rate		
	*P*	Dragonfly	Crayfish	*P*	Dragonfly	Crayfish	*P*	Dragonfly	Crayfish
*Discoglossus galganoi*	**<0.001**	**0.001**	0.727	0.766	–	–	**0.004**	**0.014**	0.886
*Pelodytes ibericus*	0.429	–	–	**<0.001**	**0.002**	0.374	**0.001**	**0.002**	0.950
*Bufo bufo*	**0.001**	**0.001**	**0.006**	**<0.001**	**<0.001**	**0.003**	**0.019**	**0.031**	0.995
*Bufo calamita*	0.053	–	–	0.110	–	–	0.254	–	–
*Hyla arborea*	**0.044**	0.737	**0.043**	0.067	–	–	**0.002**	**0.031**	**0.002**
*Hyla meridionalis*	0.118	–	–	**0.007**	**0.030**	0.746	0.120	–	–
*Pelophylax perezi*	0.957	–	–	0.054	–	–	0.067	–	–
*Alytes cisternasii*	**<0.001**	0.834	**<0.001**	**0.022**	0.177	**0.018**	**<0.001**	0.322	**<0.001**
*Pelobates cultripes*	**0.012**	0.621	**0.012**	0.838	–	–	0.640	–	–

Four species altered their life-history traits in the presence of fed crayfish (*A. cisternasii*,*B. bufo*,*H. arborea,* and *P. cultripes*; Table 3). From these species, only *B. bufo* and *H. arborea* also modified their life history when reared with fed dragonfly larvae, the responses on the two predator environments being very similar, but with some small differences. When reared with fed crayfish, *B. bufo* did not alter growth rate (while reducing time to and mass at metamorphosis) and *H. arborea*, in addition to increasing growth rate, also reduced time to metamorphosis (Table 3; Fig. 2). Tadpoles of *A. cisternasii* and *P. cultripes* reduced time to metamorphosis when exposed to fed crayfish. Finally, tadpoles of *A. cisternasii* grew faster and attained higher masses (Table 3; Fig. 2).

### Integration of responses

None of the paired trait correlations was statistically significant for *B. calamita*,*P. cultripes* and *P. perezi* (Table 4). Although there were generally relatively few significant trait correlations per species, many more and generally stronger significant associations were found in tadpoles reared with fed dragonfly larvae than with fed crayfish (19 vs. 7, Table 4). For antipredator responses induced in the presence of fed dragonfly larvae, six species (of eight responding) showed significant correlations between different defensive traits, while only two species (of four) had significant trait correlations in the presence of crayfish. Three species showed a high level of phenotypic integration (large number of significant trait correlations) when exposed to the dragonfly larvae (*D. galganoi*,*P. ibericus,* and *B. bufo*), while only one showed this in the presence of the fed crayfish (*B. bufo*; Table 4).

**Table 4:** Pearson correlations (*r* and *P*-values) performed between morphological, behavioral, and life-history traits for the nine anuran species. *N *=* *10 for all correlations. Correlations for which *P *>* *0.05 are marked as nonsignificant (N.S.). Time met – time to metamorphosis; Mass met – mass at metamorphosis; Morphol – Morphology; Behav – Behavior.

	Time met-Morphol		Mass met-Morphol		Growth rate-Morphol		Time met-Behav		Mass met-Behav		Growth rate-Behav		Time met-Mass met		Behav-Morphol	
Dragonfly	Crayfish	Dragonfly	Crayfish	Dragonfly	Crayfish	Dragonfly	Crayfish	Dragonfly	Crayfish	Dragonfly	Crayfish	Dragonfly	Crayfish	Dragonfly	Crayfish
*Alytes cisternasii*	N.S.	N.S.	N.S.	N.S.	N.S.	N.S.	N.S.	N.S.	N.S.	***r* = −0.687**	N.S.	N.S.	N.S.	***r* = −0.827**	***r* = −0.819**	N.S.
										***P* = 0.028**				***P* = 0.003**	***P* = 0.004**	
*Discoglossus galganoi*	***r* = 0.882**	N.S.	N.S.	N.S.	***r* = −0.787**	N.S.	***r* = −0.639**	N.S.	N.S.	N.S.	N.S.	N.S.	N.S.	N.S.	***r* = −0.789**	N.S.
	***P* = 0.001**				***P* = 0.007**		***P* = 0.047**								***P* = 0.007**	
*Pelobates cultripes*	N.S.	N.S.	N.S.	N.S.	N.S.	N.S.	N.S.	N.S.	N.S.	N.S.	N.S.	N.S.	N.S.	N.S.	N.S.	N.S.
*Pelodytes ibericus*	N.S.	N.S.	***r* = 0.741**	N.S.	***r* = 0.853**	N.S.	N.S.	N.S.	***r* = −0.820**	N.S.	***r* = −0.889**	N.S.	N.S.	N.S.	***r* = −0.637**	N.S.
			***P* = 0.014**		***P* = 0.002**				***P* = 0.004**		***P* = 0.001**				***P* = 0.047**	
*Bufo bufo*	***r* = −0.907**	***r* = −0.765**	***r* = −0.861**	N.S.	N.S.	N.S.	***r* = 0.849**	***r* = 0.708**	***r* = 0.871**	***r* = 0.728**	N.S.	N.S.	***r* = 0.875**	***r* = 0.803**	***r* = −0.907**	***r* = −0.635**
	***P*< 0.001**	***P* = 0.010**	***P* = 0.001**				***P* = 0.002**	***P* = 0.022**	***P* = 0.001**	***P* = 0.017**			***P* = 0.001**	***P* = 0.005**	***P*< 0.001**	***P* = 0.048**
*Bufo calamita*	N.S.	N.S.	N.S.	N.S.	N.S.	N.S.	N.S.	N.S.	N.S.	N.S.	N.S.	N.S.	N.S.	N.S.	N.S.	N.S.
*Hyla arborea*	N.S.	N.S.	N.S.	N.S.	N.S.	N.S.	N.S.	N.S.	N.S.	N.S.	***r* = −0.830**	N.S.	N.S.	N.S.	N.S.	N.S.
											***P* = 0.011**					
*Hyla meridionalis*	N.S.	N.S.	N.S.	N.S.	N.S.	N.S.	N.S.	N.S.	***r* = −0.700**	N.S.	N.S.	N.S.	N.S.	N.S.	***r* = −0.887**	N.S.
									***P* = 0.024**						***P* = 0.001**	
*Pelophylax perezi*	N.S.	N.S.	N.S.	N.S.	N.S.	N.S.	N.S.	N.S.	N.S.	N.S.	N.S.					

Concerning phenotypic alterations in tadpoles reared with fed dragonfly larvae, *D. galganoi* showed a negative correlation between activity level (behavior) and morphology, induced morphology being correlated with longer larval period (as was also activity) and reduced growth rate. *D. galganoi* was the only species for which there was a negative correlation between morphology and growth rate (Table 4). On the contrary, for *P. ibericus,* there was a positive correlation between these two traits; tadpoles that grew faster (and had larger mass at metamorphosis) also had deeper headbody and tail fin. In both these species and *H. meridionalis*, induced morphology was associated with low activity level which, in turn, was associated with higher mass (and in the case of *P. ibericus* with higher growth rate, Table 4). Activity level was also negatively correlated with growth rate in *H. arborea*.

For species showing correlations in defensive traits when reared with fed crayfish, *A. cisternasii* exhibited different relationships than the ones when reared with the fed dragonfly. In the former case, there was a negative correlation between activity and mass and between time to and mass at metamorphosis, whereas in the latter case, behavior and morphology were negatively correlated (Table 4). *B. bufo* was the only species showing a very similar number of correlations and pattern of responses integration when exposed to the native and the exotic predators, although these were always weaker for crayfish. Induced morphology was negatively correlated with both time to and mass at metamorphosis (the latter only for dragonfly), while activity was positively correlated with these two metamorphic traits. There was also a positive correlation between time to and mass at metamorphosis and a negative correlation between activity and morphology (Table 4).

## Discussion

We found that both morphological plasticity and life-history plasticity in response to predation environments were widespread in the studied community of larval anurans. As predicted, plastic responses were more common when tadpoles were exposed to the fed dragonfly than to the fed crayfish. Moreover, the responses elicited in the presence of the fed crayfish, when present, were often qualitatively different from the responses elicited in the presence of the fed dragonfly. Furthermore, also following our predictions, the integration of responses was overall higher for tadpoles reared with fed dragonfly than with fed crayfish.

Most of the studied species (all but *B. calamita*) showed phenotypic plasticity when reared with the fed dragonfly, but only four showed plasticity when reared with the fed crayfish (*A. cisternasii*,*B. bufo*,*H. arborea,* and *P. cultripes*). In larval anurans, previous studies have reported morphological and life-history responses to chemical cues from both caged dragonfly (e.g., Relyea 2001) and crayfish predators (e.g., Nyström and Åbjörnsson 2000) feeding on tadpoles. Differences between anuran species modifying or not their morphology and life history when reared with the crayfish can potentially be explained by their different ecological features. For instance, species inhabiting permanent water bodies (in our study *A. cisternasii*,*B. bufo*,*H. arborea,* and *P. perezi*) are more likely to face higher predator abundance and diversity than species from ephemeral habitats, which may select for antipredator defenses towards a wide array of predators (Richter-Boix et al. 2007). In fact, three of the four species typically reproducing in permanent habitats modified their morphology and/or life history when reared with the fed crayfish (*A*. *cisternasii*,*B. bufo,* and *H. arborea*). Further, the use of general chemical alarm cues released by consumed conspecifics to assess predation risk may have, as predicted, facilitated responses to the new predator, because two of these species (*A. cisternasii* and *B. bufo*) indeed responded quite strongly when reared with the fed crayfish (see also Nunes et al. 2013).

In this study, a higher proportion of the species responding to a specific predator treatment altered morphology in the presence of fed dragonflies (eight in eight for dragonfly against one in four for crayfish) and life-history traits in the presence of fed crayfish (four in four for crayfish against five in eight for dragonfly). This suggests that life-history characters are more readily altered in response to new selective pressures than morphological characters. Alternatively, if life-history alterations are more costly than morphological ones (Higginson and Ruxton 2010), they may be selected against when coevolutionary history between predator and prey is longer. On the other hand, crayfish recognition may be only partial and not enough to induce specific antipredator morphological defenses. In fact, among the species that altered traits in the presence of the two predators, only *B. bufo* exhibited the same type of defenses, while both *A. cisternasii* and *P. cultripes* had completely dissimilar responses, altering only morphology in the presence of dragonfly and only life-history traits in the presence of crayfish. Further studies testing the efficacy of these responses against the two different predators would be highly valuable (e.g., Gómez-Mestre and Díaz-Paniagua 2011).

### Morphological responses

The presence of fed dragonfly larvae strongly affected tadpole morphology, with tadpoles of most species developing deeper headbodies and tails and a more anterior insertion of the tail fin to the headbody. The development of deeper tails when exposed to chemical cues from predatory dragonflies is a widespread response in larval anurans (e.g., Relyea 2001; Van Buskirk 2009) that enhances the probability of survival under predation risk (e.g., Dayton et al. 2005; Gómez-Mestre and Díaz-Paniagua 2011). A more anterior insertion of the tail fin into the upper part of the headbody has also been previously reported as a response to predation risk (Van Buskirk 2009). In our study, five species (*A. cisternasii*,*D. galganoi*,*H. meridionalis*,*P. ibericus,* and *P. perezi*) developed deeper headbody regions when exposed to fed dragonfly. This may not have a direct defensive function, but may enable tadpoles to increase gut surface area and allow for lower foraging activity (Van Buskirk 2009).

The only species for which we detected morphological plasticity when reared with fed crayfish was *B. bufo*. Previous studies have generally failed to detect predator-induced morphological plasticity in this species (e.g., Lardner 2000; Richter-Boix et al. 2007). However, using also geometric morphometrics tools, Van Buskirk (2009) recently reported the same morphological alteration that we found for this species in predator presence (longer headbodies). This may indicate that morphological alterations in *B. bufo* may be subtle and require highly sensitive morphometric tools to be detected. However, defensive strategies in bufonids are known to rely mainly in unpalatability and the production of bufotoxins in the skin (Denton and Beebee 1991). Besides, the detected morphological changes are probably ineffective against predators, and more likely a by-product of changes in life history (e.g., reduction in larval period and mass at metamorphosis). *B. calamita* was the only species lacking morphological plasticity, which is in accordance with previous studies, and which is likely explained by the fact that this species reproduces in extremely ephemeral ponds, where no large aquatic predators usually exist (Lardner 2000; Richter-Boix et al. 2007).

### Life-history responses

Contrary to induced morphological responses, life-history alterations were extremely variable among the anuran species and the predator treatments. The large variation in life-history responses supports the view that these changes can either be a direct response to predation risk or a consequence of the development of behavioral and/or morphological inducible defenses (Steiner 2007). The most frequent alterations were found in time to metamorphosis and the least frequent in mass at metamorphosis. This suggests less plasticity in the latter trait, possibly because mass at metamorphosis is a crucial life-history attribute in determining adult fitness in amphibians, and because a developmental threshold (minimum size) has to be attained before metamorphosis can occur (Relyea 2007; Higginson and Ruxton 2009). *P. perezi* and *B. calamita* were the only species lacking predator-induced plasticity in life-history traits.

*Bufo bufo* tadpoles reduced both time to and mass at metamorphosis when reared in the presence of fed dragonfly and crayfish. This suggests a generalized response towards predators in *B. bufo*, likely due to a response to alarm cues, which may facilitate responses to novel predators (Semlitsch and Gavasso 1992; Sih et al. 2010; Nunes et al. 2013). Metamorphosing earlier is a rare but potentially advantageous response to predators, which has also been reported in other bufonids (Relyea 2007). Contrary to *B. bufo*, tadpoles of *D. galganoi* exposed to a dragonfly predator significantly prolonged time to metamorphosis, most likely due to eliciting strong morphological and behavioral defenses, as has been shown earlier for this species (Nicieza et al. 2006). A longer larval period can be detrimental for ephemeral pond species such as *D. galganoi* because it is likely to increase mortality when the pond dries. Morphological defenses were negatively correlated with growth, suggesting that they were more costly than behavioral defenses (see also Van Buskirk 2000).

Three of our study species increased growth rates in the presence of either fed dragonfly (*P. ibericus* and *H. arborea*) and/or fed crayfish (*A. cisternasii* and *H. arborea*). Increased growth is not a common prey response to caged predators, but it has been previously reported in amphibians (e.g., Werner 1991; Peacor 2002; Teplitsky et al. 2003; Urban 2007a) and other taxa (e.g., Spitze 1992; Johansson and Andersson 2009). It may be linked to physiological changes in metabolism, such as increased food assimilation or food conversion efficiency (McPeek 2004; Stoks et al. 2005; Steiner 2007; Dmitriew 2011; Orizaola et al. 2014). If tadpoles reach larger size through these mechanisms, this may act as a defense against size-limited predators (Urban 2007b; Dmitriew 2011). In fact, Beckerman et al. (2007) proposed that size-selective predation may induce direct physiologically mediated alterations in life-history traits of prey. Although dragonflies and crayfish are not strictly gape-limited predators, dragonflies are less able to capture and handle prey above a certain size (Werner and McPeek 1994). Increased growth rates may also be achieved by increased foraging effort and activity (McPeek 2004; Urban 2007a), which was not the case here (see Table 4). On the contrary, in *A. cisternasii* and *P. ibericus,* we found a negative correlation between behavior and mass at metamorphosis and/or growth rate, suggesting that less active animals grew faster and became larger. While this contrasts with the classical behaviorally mediated growth/predation risk trade-off (e.g., Lima and Dill 1990; McPeek 2004), other studies have also found an association between reduced activity and higher growth rates in predator presence. Johansson and Andersson (2009) proposed that the reduced activity of crucian carp in the presence of a pike predator allowed more energy to be saved and then allocated for growth. Our finding that exposure to predation environments can have a net positive effect on growth, even when behavioral defenses are elicited, reinforces the idea that a decoupling between behavior and growth is common (see also McPeek 2004; Stoks et al. 2005). However, accelerated growth may compromise postmetamorphic fitness by preventing allocation to other important traits or functions, such as immune defense and resistance to oxidative stress (Dmitriew 2011).

Two of the species that grew faster in predator presence also took a shorter time to reach metamorphosis (*A. cisternasii* and *H. arborea*). This was only observed in tadpoles reared with fed crayfish and may indicate the perception of a higher mortality risk in the aquatic habitat. While these concurrent alterations in time to and mass at metamorphosis in predator presence are not predicted to occur in nature by recent dynamic state-dependent models (Higginson and Ruxton 2010; but see Teplitsky et al. 2003), they can be beneficial for tadpoles avoiding crayfish predation.

### Integration of responses

The patterns of phenotypic integration varied widely across species and predator environments. Defensive phenotypes were generally more integrated for tadpoles reared with the fed dragonfly than with the fed crayfish, probably reflecting a better adaptation to the known predator.

Overall, correlations were very variable from species to species, suggesting species-specific defensive strategies. Further, the same traits were sometimes related in opposite manners in the same predator environment. For instance, considering trait alterations under the presence of the fed crayfish, for *A. cisternasii,* time to and mass at metamorphosis were negatively correlated, whereas in *B. bufo,* these traits were positively related. This shows that traits are not linearly related across species and that outcomes are specific for each prey species. Nevertheless, the correlation between behavior and morphology was consistently negative across species, indicating that a decrease in activity did not reduce the energy necessary for developing morphological defenses. On the contrary, alterations in morphology could have been indirect effects of reduced tadpole activity, as has been observed before (e.g., Johansson and Andersson, 2009). This suggests that these two types of defenses may frequently complement or augment each other, which has been shown before (trait cospecialization, sensu DeWitt et al. 1999) and is predicted to maximize fitness (DeWitt and Langerhans 2003; Steiner and Pfeiffer 2007).

## Conclusions

Our work highlights the importance of studying whole communities to evaluate responses to predators, adding to the growing evidence that different species use specific suites of defenses against the same predator. The results reinforce the notion that interspecific differences in behavioral, morphological, and physiological characteristics, as well as in species ecology, select for different antipredator responses in different species (Lardner 2000). We found that, from the nine anurans present in the study area, four species modified their morphology and/or life history when reared with a novel predator feeding on conspecifics. In three of these species, tadpoles reared with fed crayfish exhibited a different suite of responses than tadpoles reared with fed dragonfly, suggesting predator-specific responses to the invasive predator. Responses of tadpoles reared with the invasive crayfish could be generated by the recognition of this predator as a threat, or by the reaction to alarm chemical cues produced during predation on conspecifics or the combination of these with specific predator cues. In any case, the responses are likely to confer tadpoles some defense against this predator. Two species, *P. ibericus* and *P. perezi*, while showing plasticity when reared with the fed dragonfly, did not respond when reared with crayfish (a similar result was found for behavioral responses; Nunes et al. 2013), likely not recognizing the active crayfish as a threat. As such, these species seem to be undefended against *P. clarkii*, which may lead to population declines and compromise their persistence in habitats invaded by the crayfish. This acquires special relevance in the case of *P. ibericus*, a species endemic to the Iberian Peninsula (Loureiro et al. 2008). As most inland aquatic habitats in the Iberian Peninsula are already invaded by *P. clarkii*, and most of the areas not invaded seem to be suitable for its establishment (Capinha and Anastácio 2011), special efforts are needed to determine the level of threat that this invasive crayfish poses to native anurans. Examining how prey species vary in their responses to new predators is essential for understanding the dynamics of predator–prey interactions following biological invasions and, ultimately, for preventing extinctions in invaded communities.

## Supporting Information

Figure S1 Location and detailed explanation of the 20 landmarks digitized for estimating tadpole body shape.
