# Supplementary material for: Morphological and life-history responses of anurans to predation by an invasive crayfish: an integrative approach
Source: Ecol Evol. 2014 Mar 25;4(8):1491–503. doi: 10.1002/ece3.979 (PMC4020706; doi:10.1002/ece3.979)
Supplement: Figure S1 — Location and detailed explanation of the 20 landmarks digitized for estimating tadpole body shape. [file ece30004-1491-sd1.docx]

**Online Supporting Information**

Nunes, A.L., Orizaola, G., Laurila, A. and Rebelo, R. Morphological and life-history responses of anurans to an invasive predator: an integrative approach.

**Fig. S1.** Location and detailed explanation of the 20 landmarks digitised for estimating tadpole body shape.

1 - Most anterior point of the headbody

2 - Centre of the eye

3 - Point of intersection between the headbody and the upper edge of the tail fin

4 - Point of the tail fin at two thirds of the distance between landmarks 1 and 9

5 - Point of the headbody (dorsal side) at two thirds of the distance between landmarks 1 and 9

6 - Point of the headbody (ventral side) at two thirds of the distance between landmarks 1 and 9

7 - Point of intersection between the headbody and the upper edge of the tail muscle

8 - Point of intersection between the headbody and the central line of the tail muscle

9 - Point of intersection between the headbody and the lower edge of the tail muscle

10 - Point of intersection between the anus and the lower edge of the tail fin

11 - Point of the tail fin (dorsal side) at one quarter of the distance between landmarks 8 and 20

12 - Point of the tail fin (ventral side) at one quarter of the distance between landmarks 8 and 20

13 - Point of the tail fin (dorsal side) at half distance between landmarks 8 and 20

14 - Point of the tail muscle (dorsal side) at half distance between landmarks 8 and 20

15 - Point of the tail muscle (ventral side) at half distance between landmarks 8 and 20

16 - Point of the tail fin (ventral side) at half distance between landmarks 8 and 20

17 - Point of the tail fin (dorsal side) at three quarters of the distance between landmarks 8 and 20

18 - Point of the tail fin (ventral side) at three quarters of the distance between landmarks 8 and 20

19 - Tip of the tail muscle

20 - Tip of the tail fin
